# The U-shaped impact of export quality on firms’ innovation output: Empirical evidence from China

**DOI:** 10.1371/journal.pone.0298358

**Published:** 2024-02-23

**Authors:** Yueling Cai, Gongliang Wu

**Affiliations:** 1 School of Economics and Management, Zhejiang Sci-Tech University, Hangzhou, Zhejiang, PR China; 2 School of Economics, Zhejiang University of Technology, Hangzhou, Zhejiang, PR China; Universiti Malaysia Sabah, MALAYSIA

## Abstract

Ensuring product quality plays a pivotal role in the successful execution of new product development and serves as a cornerstone for fostering innovation within enterprises. In this study, we investigate the impact of export quality at the firm level on innovation outcomes, utilizing data from Chinese publicly listed companies and customs data spanning the period from 2010 to 2015. Our analysis uncovers a notable U-shaped relationship between export quality and innovation output in Chinese manufacturing enterprises, particularly with respect to invention patents and utility model patents. Subsequent exploration of heterogeneity reveals that this U-shaped relationship is consistent for non-state-owned enterprises, firms operating in high-technology sectors, young firms, firms exporting to high-income countries, firms situated in the eastern region, and firms engaged in processing trade. Additionally, we find a positively significant effect of Chinese manufacturing export quality on the innovation output of firms exclusively involved in general trade. This paper’s key contribution lies in identifying the U-shaped influence of export quality on firms’ innovation output and its applicability. The findings suggest that Chinese manufacturing enterprises need to improve the quality of their export products on a long-term and sustained basis. A series of quality standards for the dynamic development of different industries should be formulated, and actively safeguard the innovation needs of these enterprises, thereby enhancing the value chain position of Chinese manufacturing enterprises.

## 1. Introduction

In recent times, many businesses have placed significant emphasis on pursuing innovation, driven by a multitude of factors [1, 2]. Innovation has emerged as a pivotal means of gaining a competitive edge, a critical determinant of an organization’s long-term viability, and a fundamental requirement for ensuring sustainability [3, 4]. However, the traditional concept of sustained competitive advantage is no longer tenable in a highly unpredictable environment [5]. A firm’s competitiveness is closely tied to its position within the value chain. The conceptualization of quality across the value chain can be seen as an expression of influence or authority. The importance of quality becomes evident when examining the impact of “lead firms” on the division of labor and entry barriers within the chain. As highlighted by Ponte and Gibbon [6], control over the qualification of a specific product is a crucial motivating factor for leading firms in the context of quality agreements. Firms engaged in innovation have the prerogative to establish quality benchmarks.

According to Cooper and Kleinschmidt [7], the determinant of success in product innovation is contingent upon the superiority or high quality of the product, which stands as the primary factor influencing the achievement of corporate objectives. Moreover, creativity is an inherent outcome of high-quality cognitive processes [8]. Hence, it is imperative to have a more comprehensive comprehension of the principles underlying innovation and quality control. The customer and user perspectives are widely recognized as a significant driving force behind innovation and the implementation of overall quality management. When considering innovation, it is advisable to strive for the introduction of high-quality variability in response to these influential factors. Based on existing research, product quality is regarded as a key competitive advantage for a new product. Innovation can be conceptualized as an outcome of Total Quality Management and employing quality management tools and techniques can facilitate the systematic management of the product and service innovation process by executive management teams [9]. The significance of product quality lies in its role as a fundamental pillar for fostering enterprise innovation and as a crucial assurance for the successful execution of new product development initiatives. Hence, it is imperative to elucidate the correlation between product quality and innovation. Multiple research has demonstrated that there is a positive relationship between innovation and the quality of exports [2–4, 10, 11]. Numerous researchers have examined the matter of product innovation and quality through the lens of production [12–14]. Their primary emphasis revolves around Storper and Salais’ [15] concept of the “world of production” specifically exploring the transition of industries from general to specialized production. The association between the concept of quality and the decrease in process variability, specifically in terms of quality control, has been duly acknowledged [6]. For instance, the concept of overall quality management is commonly linked to the notion of standardization, which is believed to result in a decrease in the scope of innovation [1].

This research delves into the relationship between firm-level export quality and patent output within a sample of Chinese listed companies, providing novel insights that deviate from previous findings. The contributions of this study are (1) finding empirical evidence of the U-shaped effect of export product quality on innovation output in the Chinese manufacturing sector. Using a sample of Chinese listed companies, this study provides insights into the relationship between firm-level export quality and patent output. Established studies have focused on assessing how innovation affects product quality, while limited attention has been paid to exploring how product quality affects innovation [2–4, 10, 11], and have yielded inconsistent results, with both positive and negative impacts of product quality on innovation. (2) Further clarifies the scope of application of the U-shaped effect of quality of Chinese manufacturing exports on innovation output, including non-state-owned firms, young firms, high-tech firms, and firms in the eastern region. The results of these heterogeneity tests help to better understand the applicability of the findings on the impact of export quality on innovation output, and also provide practical implications for China’s manufacturing export trade. Chinese manufacturing enterprises need to improve the quality of their export products on a long-term and sustained basis. A series of quality standards for the dynamic development of different industries should be formulated, and actively safeguard the innovation needs of these enterprises, thereby enhancing the value chain position of Chinese manufacturing enterprises.

This paper is organized into five sections. Section 1 serves as the introduction. Section 2 provides a review of pertinent literature on product quality and firm innovation and establishes a hypothesis as the foundation for the empirical study in this paper. In Section 3, we detail the empirical model setting, variable selection, and data processing. Section 4 presents the empirical results and facilitates a discussion of the findings. Lastly, in the concluding section, we draw main conclusions and highlight policy implications.

## 2. Theoretical analysis and research hypothesis

### 2.1 Quality and the value chain

The evaluation of quality components is an ongoing process that considers evolving standards [6]. In various contexts, different types of rational agreements or “value orders” are employed [16]. Murdoch and Miele [12] propose that markets rely on six conventions, also known as value orders, which serve as expressions of quality standards. These industrial conventions assess the outcomes of industrial manufacturing, operational efficiency, and reliability.

Geographical and traditional production methods hold high regard within domestic traditions. The resolution of opinion conventions, such as addressing uncertainties regarding quality, is accomplished through judgments made by a “reputable professional”. Consumer recognition of trademarks and brands constitutes a fundamental aspect of public customs. Commercial conventions predominantly emphasize four key elements: price, promotion, utility, and commercial excellence. Boltanski and Thevenot [16] categorize commercial conventions as market worlds in their analysis. Commercial conventions can be broadly equated with market conventions, which outline qualitative attributes that are geographically favored.

The physical attributes of quality can be quantified and subjected to standardization. However, the social construction of quality is influenced by various behaviors, preferences, and reasons, and it depends on the interactions of producers, consumers, retailers, and other stakeholders throughout the value chain [17]. According to Ponte [18], the governance of global value chains (GVCs) can be categorized into three distinct forms. Governance, in this context, is a concept deeply rooted in normativity, which involves the process of transforming existing behaviors or attributes into established standards or norms. In the realm of innovation within product value chains, innovation encompasses the introduction of novel items and incremental advancements in current products, including modest modifications in packaging. It also involves negotiating and enhancing product quality. The distribution of products is undergoing a significant shift from traditional channels to processing companies and supermarket chains, which now place a higher premium on stability, reliable delivery, consistent high quality, and steady pricing [19]. This emerging trend may pose challenges for sectors heavily reliant on specific raw materials to meet desired product standards. To maintain a high standard of quality, it is essential to establish and adhere to consistent conventions and agreements regarding procedural norms. The producer-user interface undergoes changes when there is consensus on quality criteria. Consequently, the process of innovation relies on the integration of interactive learning across the entire value chain.

### 2.2 The impact of innovation on product quality

While prior scholarly works have addressed the topic of product innovation in oligopolistic enterprises [20, 21], there remains an unresolved issue in the market. This pertains to situations where firms across various industries place significant emphasis on their ability to innovate as a means to boost market demand. In the face of intense market competition, enterprises continuously engage in product innovation to stimulate consumer demand and encourage purchasing behavior. Monopolies, despite facing limited competition, also employ innovation strategies to diversify their offerings. Rather than provoking price-based competition, oligopolistic enterprises turn to product innovation as a means to differentiate themselves from their competitors. Therefore, the capacity to innovate has become a fundamental skill for all companies, regardless of their competitive strategies. Previous studies [22, 23] have explored the impact of competitors’ size on businesses’ adoption and diffusion of innovations. These studies suggest that the size of competitors does not have a substantial effect on the adoption and diffusion process.

Numerous studies have consistently shown a positive correlation between innovation and the quality of exports [2–4, 10, 11]. Dubey and Wu [24], Fishman and Rob [25] have presented compelling evidence illustrating the efforts exerted by manufacturing organizations to improve product quality through the adoption of costly and potentially risky innovation strategies. Koufteros et al. [20] have provided empirical support for this claim based on a study involving 244 manufacturing enterprises, reinforcing the notion that product innovation has a beneficial impact on quality. Furthermore, the research conducted by Dubey and Wu [24] has revealed that product innovation predominantly occurs within industries of moderate size and is primarily undertaken by oligopolistic enterprises seeking to enhance their product offerings through innovative practices. It’s worth noting that prior research has indicated that the enhancement of product quality does not always result directly from product innovation, as innovative products frequently encounter uncertainties during the initial stages of development [21, 26, 27].

### 2.3 The impact of quality management on innovation

The concept of product quality is commonly defined as a company’s ability to deliver products that meet customer and market expectations [28]. Current literature on quality management and competitive strategy suggests a significant relationship between product quality and product innovation. According to the findings of Lin and Lu [29], product quality relates to a product’s effectiveness in satisfying both consumer and environmental demands. Moreover, their research underscores the role of product quality in influencing product innovation. Companies actively involved in product innovation are typically responsible for producing high-quality items. Furthermore, the implementation of total quality management, which aims to enhance product quality in terms of performance, reliability, and durability, can significantly boost an organization’s innovation capacity.

Looking at the broader context of process management, total quality management undeniably plays a vital role in product innovation management. Among the various categories of innovation, four have garnered significant attention from scholars: administrative and technological innovation, product and process innovation, radical and incremental innovation, and product and service innovation. Bossink [30] conducted a study in the Netherlands construction industry, uncovering a potential link between quality management and innovation management. This finding is consistent with previous research conducted in various industries, as reported by Keogh and Bower [31]. Nonetheless, this research does not provide empirical evidence to support the notion that product quality fosters product innovation. While the literature extensively establishes the influence of product quality on product innovation, its impact on other forms of innovation remains incompletely understood and necessitates further investigation [29].

### 2.4 The impact of export quality on innovation

Companies with superior product quality in their exports typically have a stronger foundation in terms of human and financial resources. By engaging in international trade through exports, these firms effectively tap into international customer feedback, gather valuable knowledge and technology from abroad, and thereby enhance their innovation performance, reaping multiple benefits [32]. However, in this process, novice, sales-driven organizations entering the global market for the first time may encounter challenges in quickly comprehending, assimilating, and leveraging information, in contrast to well-established enterprises. This may hinder their ability to promptly translate information into innovative outcomes, impeding their short-term international competitiveness. Nevertheless, the long-term adaptability and development of these firms, as well as their capacity to cultivate significant competitiveness, depend on their unique circumstances [33].

The presentation of innovation output involves an accumulation process. When an enterprise expands its presence in the domestic or international market, it initially requires substantial effort to adapt, learn, and master new dynamics [34]. Consequently, there may be a temporary decrease in innovation output compared to the initial level. However, as the enterprise improves the quality of its exported products, it enhances its resource accumulation and learning capabilities, leading to a further enhancement of innovation output [35].

China’s manufacturing export product quality has markedly improved, owing to the inflow of foreign capital that has introduced new production lines, production technology, management practices, and high-quality intermediate products [36]. These investments have led to varying degrees of technological spillover, resulting in a continuous enhancement of the quality of China’s export products. Furthermore, this influx has spurred the gradual expansion of production scale and introduced new perspectives and concepts to domestic enterprises.

Despite these positive developments, challenges related to promoting innovation and achieving independent innovation may deter some enterprises from prioritizing these endeavors, temporarily affecting their innovative output. In contrast, some firms may prioritize increasing their market share over innovation, leading to a temporary decline in their innovative output [37]. However, as competition among enterprises intensifies, traditional battles centered on price and quality will evolve into a new form of competition that places a greater emphasis on innovation. Enterprises will gradually recognize the limitations of traditional competition methods and understand that sustainability can only be achieved through innovation. It is through innovation that organizations can make significant advancements in the face of fierce competition, thus enhancing their innovation productivity [38].

Enhancing the quality of export products can be categorized into two primary approaches. The first approach involves internal research and development (R&D) innovation within the enterprise, encompassing improvements in production processes, technology, and quality management [32]. These efforts are directed toward enhancing the quality of products intended for export. The second approach involves the acquisition of high-quality intermediate goods, followed by their processing and assembly, leading to subsequent export. This approach may also encompass the adoption of foreign management techniques or patented technologies, facilitating the implementation of novel production processes or assembly lines for the production of superior-quality products. Notably, an increase in the utilization of the second approach by domestic firms to enhance the quality of their export products may lead to a decrease in the firms’ patent output [39]. Consequently, it can be inferred that the utilization of advanced technology by domestic firms, achieved through direct introduction rather than independent invention, to improve product quality has a restraining effect on the innovation output of these firms, thus impeding the enhancement of export product quality. Over time, domestic enterprises possess the capability to consistently assimilate the external knowledge and technology, resulting in a gradual augmentation of their innovative outcomes [40]. Building on the information presented above, we propose the following hypothesis.

#### Hypothesis

Export quality exhibits a U-shaped effect on the innovation outputs of Chinese manufacturing enterprises.

## 3. Model setting, variable selection, and data processing

### 3.1 Model setting

Based on the theoretical analysis and research hypothesis outlined above, and drawing from existing literature, we construct the empirical model for the impact of export quality on the innovation output of Chinese manufacturing enterprises as follows:

$${patents}_{i,t}=\alpha +\beta 1{expqlt}_{i,t}+\beta 2{expqlt}_{i,t}^{2}+\Sigma \gamma k{control}_{i,t}+\delta_{i}+\delta_{t}+\delta_{s}+\delta_{p}+\varepsilon_{i,t}$$ (1)

Among them, *patents*_*i*,*t*_ represent enterprise innovation output, subscripts *i* and *t* represent company and year respectively, *expqlt*_*i*,*t*_ represent export quality, ${expqlt}_{i,t}^{2}$ represent the square term of export quality, Σγ_k_*control*_*i*,*t*_ represent a set of a series of control variables, α is a constant term, β_1_ and β_2_ represent the regression coefficients of export quality and export quality square terms, γ_k_ represents the coefficient of each control variable, δ_i_ represents the individual effect of the enterprise, δ_t_ represents the annual effect, δ_s_ represents the fixed effect of the industry, and δ_p_ represents the fixed effect of the province, ε_i,t_ are random disturbance terms. In addition, due to the certain time lag in the display of enterprise innovation output, in the empirical research below, the lag period of the export quality of the explanatory variable will also be taken into consideration based on specific circumstances. This approach can also alleviate the endogeneity problem. The downside is that the creation of lagged variables will lose the number of samples.

In our specific analysis methods, we observe that the explained variable *patents* in the empirical model consists of count data. Notably, its variance significantly surpasses its mean, indicating over-dispersion, as shown in Table 1. However, given that the minimum value is 2 and there is no evidence of zero inflation, we recommend employing the Panel Negative Binomial Model for our regression analysis.

**Table 1:** Statistical descriptions.

Variable name	obs	mean	Std deviation	min	max
***expqlt***	2,044	0.000	1.000	-2.310	1.952
***patents***	1,054	104.1	256.2	2	1,880
***invention***	924	32.93	75.12	0	528
***utility***	571	24.11	48.56	0	309
***design***	467	8.773	22.44	0	141
***domestic***	1,054	102.8	253.5	1	1,880
***overseas***	1,054	0.798	4.681	0	41
***rdspendsum***	2,411	1.374	2.807	0.057	20.17
***rdspendsumratio***	2,373	0.495	3.708	0.110	24.18
***rdperson***	1,031	542.1	944.6	30	7,028
***xr***	2250	4.268	13.41	0.016	111.4
***trf***	2146	0.292	1.834	1.1e-05	6.013
***finance***	2,645	0.179	0.124	0.017	0.760
***liability***	2,646	0.380	0.198	0.045	0.826
***return***	2,646	0.066	0.055	-0.110	0.240
***age***	2,639	1.447	0.533	0.358	3.042

### 3.2 Variable selection

#### 3.2.1 Dependent variables

This article employs a company’s count of patent applications as a proxy variable for the company’s innovation output, a methodology established by prior studies [41, 42]. The patent system operates as a legal framework that safeguards inventions and innovations, actively fostering technological advancement [43]. It is crucial to note that there exists a considerable time lag from the initial patent application to its authorization. The patent authorization process entails several stages, including preliminary examination, municipal evaluation, and final authorization, which typically takes between 2 to 4 years [44]. In order to avoid the impact of the approval cycle, this article decided to use the number of patent applications to measure innovation output. *Invention*, *utility*, and *design* respectively represent the number of applications for invention patents, utility model patents, and design patents. *Domestic* and *overseas* represent the number of domestic and foreign patent applications respectively. ln*patents* is the total number of patent applications after taking the natural logarithm, *rdspendsum*, *rdspendsumratio*, and *rdperson* respectively represent R&D investment, R&D investment proportion, and the number of R&D personnel.

#### 3.2.2 Independent variables

The calculation of export product quality *expqlt* at the enterprise level draws on the practice of Khandelwal [45], Amiti & Khandelwal [46], and Nevo [47]. The main logic is as follows:

$$ln{exp\_quantity}_{i,p,c,t}=\sigma ln{exp\_price}_{i,p,c,t}+\varphi_{p}+\varphi_{ct}+\varepsilon_{i,p,c,t}$$ (2)

Among them, ln*exp_quantity*_*i*,*p*,*c*,*t*_ represent the export quantity of the same company i, the same year *t*, the same product *p*, and the same country *c* (take the natural logarithm), ln*exp_price*_i,p,c,t_ represent the same company, the same year, The export price of the same product and exported to the same country (take the natural logarithm), σ is the elasticity of substitution between products, *φ*_p_ and *φ*_*ct*_ represent the control on the product, country and time levels respectively, *ε*_*i*,*p*,*c*,*t*_ is a residual item that contains product quality information. The residual items *ε*_*i*,*p*,*c*,*t*_ can represent the factors that affect the product sales after excluding the price factor, that is, the product quality. Further, standardize *ε*_*i*, *p*, *c*, *t*_, and then use the ratio of the export value at the “enterprise-year-product-country” level and the “enterprise-year-product” level as the weight, and weight the product quality at the national level. After that, the product-level quality is obtained. Finally, the weighted sum of the product-level quality is carried out according to the ratio of the export value at the “enterprise-year-product” level and the “enterprise-year” level as the weight, and finally, the export quality at the enterprise level is obtained as *expqlt*. *expqlt*^2^ represents the square term of the export quality after standardization.

#### 3.2.3 Control variables

The age of the company *age* is represented by the difference between the year of the current year and the year of establishment plus one. The company may have different performances in strategic decision-making in different growth periods, so it is controlled [48]. Enterprise R&D investment *rdspendsum*, as a key resource for innovation, provides an innovation foundation for the enterprise, represents the overall innovation capability of the enterprise, and determines the innovation output to a large extent, which must be controlled. Commercial credit financing capacity *finance* is expressed by the ratio of company accounts payable to sales revenue [49]. The asset-liability ratio *liability* is expressed by the ratio of total liabilities to total assets. The debt-to-asset ratio can control the innovation output of the enterprise from the perspective of the capital structure of the enterprise [50]. The return on invested capital *return* is expressed as the ratio of pre-interest and after-tax operating profit to invested capital.

#### 3.2.4 Instrumental variables

In our model, we employ exchange rate *xr* and tariff *trf* as instrumental variables to address endogeneity in our explanatory variables related to export quality. This issue typically arises from two primary sources: the omission of relevant variables and mutual causality. To tackle the former concern, we’ve incorporated an extensive set of control variables into our model, along with four fixed effects, namely enterprise, year, industry, and province, for meticulous control.

To address potential endogeneity stemming from mutual causality, we employ the instrumental variable method to rectify the regression results. Our chosen instrumental variables are real exchange rates and tariffs. It’s noteworthy that following China’s accession to the World Trade Organization (WTO), the country experienced a substantial reduction in tariffs, as documented by Lu and Yu [51]. Both exchange rates and tariffs are exogenous variables, exerting a significant influence on companies’ trade-related decision-making.

Specifically, the real exchange rate represents the ratio of the Chinese Renminbi (RMB) to foreign currency and is sourced from the Penn World Table within the University of Pennsylvania World Database. Tariff data, on the other hand, is extracted from the United Nations Commodity Trade Database (UN Comtrade). To align the tariff data with our customs database, we match it with connection variables such as year, country, and industry. Subsequently, we compute enterprise-level tariff data. A similar procedure is followed for exchange rate data, involving the matching of year and country connection variables with our customs database, culminating in enterprise-level exchange rate data.

### 3.3 Data processing

Due to the lack of the two indicators of product unit price and product quantity in the original data of the customs database in 2016, it is impossible to infer 2016 product quality data at the enterprise level through the demand inference method. Therefore, by merging the 2010–2015 RESSET customs database and the overseas direct investment database of CSMAR, there are a total of 2646 observations and a sample of 818 manufacturing companies after the merger. The combined data is an unbalanced panel.

The existence of outliers will bias the regression results, so the winsorize treatment is implemented for all continuous variables at the 1% and 99% quantiles, while also ensuring that the sample size is not reduced. The descriptive statistics of the variables are shown in Table 1. In order to avoid too large differences in the order of magnitude between the variables, the company’s age measurement unit is changed to ten years, the R&D expenditure measurement unit is changed to 100,000 yuan, and the R&D investment measurement unit is changed to 100 million yuan. In addition, the export product quality *expqlt* and its square term *expqlt*^*2*^ are statistical results after standardized processing, to avoid the collinearity problem that will occur in the regression later.

In Table 2, we present the correlation coefficient matrix of the primary variables. Notably, the correlation coefficients between each of the explanatory variables are all below 0.5, indicating the absence of substantial collinearity among them. Consequently, these variables can be included in the same regression model. From an initial examination of the first column, we observe that the correlation coefficient between export quality and innovation output stands at -0.125, signifying statistical significance at the 1% level. Likewise, the correlation coefficient between the square term of export quality and innovation output is 0.138, also significant at the 1% level. This initial analysis provides some support for our hypothesis. As for the correlations between the age of enterprises and trade patterns and the dependent variables, they do not exhibit significant relationships. However, most of the remaining primary explanatory variables demonstrate statistically significant correlations with the dependent variables, typically at the 5% level.

**Table 2:** Correlation coefficient matrix.

	*patents*	*expqlt*	*expqlt* ^*2*^	*age*	*finance*	*liability*	*return*	*property*	*trdcd*	*rdspendsum*
***patents***	1									
***expqlt***	-0.125***	1								
***expqlt*** ^***2***^	0.138***	-0.316***	1							
***age***	-0.0003	0.022	-0.012	1						
***finance***	0.047**	-0.229***	0.136***	0.079***	1					
***liability***	0.211***	0.054***	0.029*	0.277***	0.283***	1				
***return***	0.092***	-0.058***	-0.039**	-0.073***	-0.210***	-0.172***	1			
***property***	-0.160***	0.037**	0.0009	-0.149***	-0.090***	-0.352***	0.089***	1		
***trdcd***	0.043	0.169***	-0.132***	-0.103***	-0.068***	0.059***	0.038**	-0.037***	1	
***rdspendsum***	0.667***	-0.061***	0.097***	0.032***	0.089***	0.253***	0.060***	-0.250***	0.0250	1

Note:*** p<0.01

** p<0.05

* p<0.1.

## 4. Empirical results and discussion

### 4.1 Baseline regression

**Fig 1:**
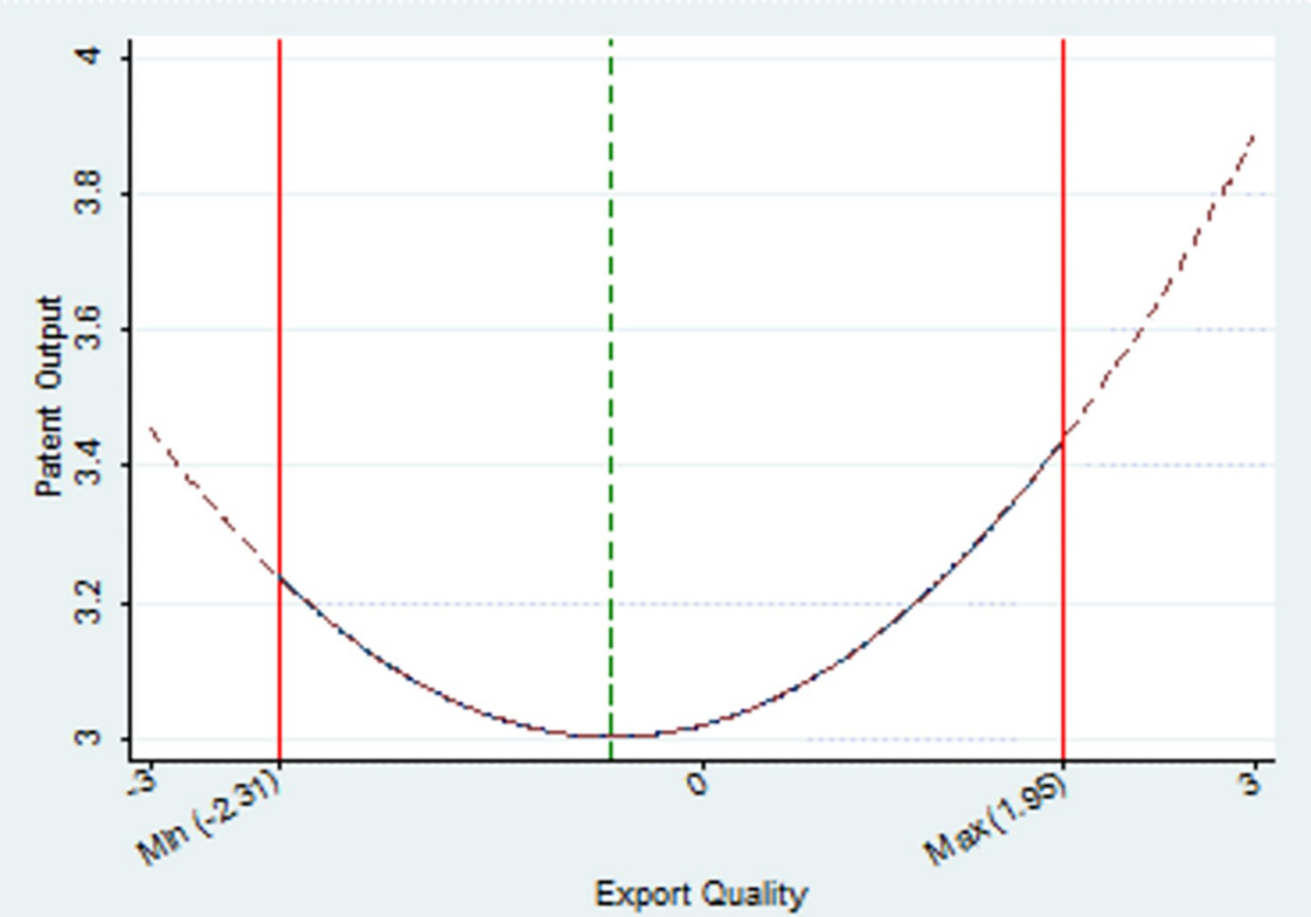
The U-shaped effect of export quality on innovation output.

Table 3 presents the baseline regression results assessing the impact of export quality on firm innovation output. The findings, captured in Model (1) aligned with measurement model (1), reveal a significant influence of the square term of export quality on enterprise innovation output. Specifically, the coefficient for this term is 7.251, reaching statistical significance at a 5% confidence level. These results suggest a notable U-shaped relationship between export quality and innovation production. Initially, an improvement in export quality leads to a reduction in innovation output, followed by an increase. After conducting calculations, we pinpoint the nadir of this U-shaped relationship at -0.1. The independent variable’s values span a range from -2.31 to 1.95, encompassing this inflection point. Notably, there are 366 observations on one side of the symmetry axis and 465 on the other, affirming the presence of a U-shaped pattern. For a visual representation (Fig 1), where the dependent variable, innovation output, is log-transformed. The green dashed line signifies the symmetry axis of the fitted curve, while the vertex marks the inflection point. The red solid lines on either side indicate the ranges of values for the independent variable, export quality, across the sample period.

**Table 3:** Baseline regression.

Variable	(1)	(2)	(3)	(4)	(5)	(6)
	*patents*	*invention*	*utility*	*design*	*domestic*	*overseas*
***expqlt***	0.021 (0.424)	0.060 (1.083)	0.071 (0.925)	-0.172 (-1.387)	0.036 (0.702)	
***expqlt*** ^***2***^	7.251** (2.152)	8.370** (2.194)	1.303** (2.265)	-1.300 (-1.359)	8.251** (2.225)	
***L*.*expqlt***						0.062 (0.335)
***L*.*expqlt*** ^***2***^						0.337** (2.488)
**Cons**	2.868*** (8.889)	2.276*** (6.370)	1.411*** (2.878)	-0.353 (-0.433)	2.730*** (7.822)	-2.328* (-1.647)
**Obs**	831	744	433	396	831	826
**Number of companies**	368	328	228	215	368	348
**Control Variables**	Yes	Yes	Yes	Yes	Yes	Yes
**Year FE**	Yes	Yes	Yes	Yes	Yes	Yes
**Company FE**	Yes	Yes	Yes	Yes	Yes	Yes
**Industry FE**	Yes	Yes	Yes	Yes	Yes	Yes
**Province FE**	Yes	Yes	Yes	Yes	Yes	Yes
**Likelihood ratio test**	669.2	590.51	175.21	100.9	657.63	21.12

Note:*** p<0.01

** p<0.05

* p<0.1. Values in parentheses below the regression coefficients are z values.

One plausible explanation for these results pertains to the prevalence of numerous processing trade enterprises experiencing substantial growth in trade volume within the country. These firms specialize in straightforward production tasks, allowing them to generate labor income rapidly and achieve economic gains. Consequently, a substantial presence of processing trade activities within the country contributes to improving export product quality. This is achieved through the utilization of large-scale, standardized production lines during the production process. However, it’s unlikely that simple and repetitive production tasks can stimulate enterprise innovation. This initially leads to a crowding-out effect on innovation output. As product quality reaches a certain threshold, organizations must shift their focus towards further growth and advancement through independent research and development (R&D) and innovation. During this phase, firms are expected to intensify their innovation efforts, resulting in a subsequent increase in overall innovation output.

Patents can be categorized into three types based on their nature: Patents for inventions, utility model patents, and design patents. In column (2), the data reveal a U-shaped relationship between the quality of exports and the number of invention and utility model patents filed by firms. As export quality improves, the number of patents initially decreases, reaching a minimum point at -0.4, and then starts to increase. This dataset comprises 275 observations on one side of the first inflection point and 469 observations on the other side. In column (3), we find a U-shaped effect of export quality on utility model patents, with the inflection point observed at -0.3. This dataset includes 160 observations on one side of the inflection point and 273 on the other. Column (4) shows that export quality has no statistically significant impact on the output of design patents. Further heterogeneity tests, considering the type of innovation output by enterprises, suggest that as export quality improves, there is an initial decrease followed by an increase in the number of applications for invention patents and utility model patents. This indicates that, in the long term, improving export quality can lead to more substantial, practical, and higher-content innovation outputs for enterprises.

Based on the geographical location of patent applications, they can be categorized into domestic patents and international patents. In column (5), a U-shaped relationship is observed between the influence of export quality and the number of domestic patent applications, with the inflection point at -0.2. There are 334 observations on the left side of the inflection point and 497 on the right side. Column (6) demonstrates a U-shaped lag effect of export quality on the quantity of international patent applications filed by firms, with the inflection point determined to be -0.1. There are 295 observations on the left side and 531 on the right side of the inflection point. These heterogeneity tests based on patent application location reveal that the impact of improved export quality on firms’ innovation outputs exhibits a U-shaped pattern characterized by initial inhibition followed by subsequent promotion. All of these relationships have been found to be statistically significant at the 5% level. Additionally, the effect on domestic patent outputs is observed to occur more promptly.

### 4.2 Robustness tests

The results of the robustness tests are presented in Table 4. The procedure involves the substitution of the indicators for the dependent variable. The regression analysis in column (1) demonstrates the outcome when the dependent variable is substituted with its natural logarithm. Notably, the regression coefficient for the squared term of export quality is 5.322, indicating statistical significance at the 5% level. The findings suggest that there exists a noteworthy U-shaped relationship between the quality of exports and the innovation output of enterprises. The inflection point of the U-shaped relationship is determined to be -0.3, with a sample size of 306 observations on one side and 525 observations on the other side. The second column presents the regression outcome obtained by substituting the dependent variable with the count of research and development (R&D) workers. The analysis reveals a U-shaped relationship, with a computed turning point of -0.1. The number of observations on either side of this turning point is 331 and 333, respectively. Columns (3) and (4) employ firms’ research and development (R&D) spending as the dependent variable. The regression results include the inclusion of explanatory variables such as export quality and its squared term, as well as the lagged one-period export quality and its squared term. The regression analysis of model (3) reveals a U-shaped relationship with a turning point at 1.1. The dataset consists of 2933 observations on one side of the turning point and 429 observations on the other side. The regression results for model (4) indicate that the U-shaped connection reaches its turning point at 0.8. The number of observations on either side of the turning point is 2103 and 813, respectively. Column 5 displays the regression outcomes subsequent to substituting the dependent variable with the ratio of R&D investment made by the enterprises. The inflection point of the U-shaped correlation is determined to be 0.6, with a sample size of 1870 observations on one side and 804 observations on the other side. The regression results from model (2) through model (5) demonstrate that there continues to be a substantial U-shaped association between export quality and firms’ innovation output. This section suggests that the aforementioned regression results are robust.

**Table 4:** Robustness test.

variable	(1)	(2)	(3)	(4)	(5)
	*lnpatents*	*rdperson*	*rdspendsum*	*rdspendsum*	*rdspendsumratio*
***expqlt***	0.074 (1.641)	0.022 (0.508)	-18.48** (-2.570)		-0.142** (-2.422)
***expqlt*** ^***2***^	0.077** (2.350)	0.648** (2.055)	8.186* (1.668)		0.115*** (2.891)
***L*.*expqlt***				-20.22** (-2.271)	
***L*.*expqlt*** ^***2***^				12.27** (1.983)	
**Control variables**	Yes	Yes	Yes	Yes	Yes
**Obs**	831	1235	3,362	2,916	2,674
**Number of companies**	348	675	716	676	706
**Year FE**	Yes	Yes	Yes	Yes	Yes
**Company FE**	No	Yes	No	No	No
**Industry FE**	Yes	Yes	Yes	Yes	Yes
**Province FE**	Yes	Yes	Yes	Yes	Yes
**Likelihood ratio test**		222.2			
**R** ^**2**^	0.405		0.555	0.573	0.521

Note:*** p<0.01

** p<0.05

* p<0.1. Values in parentheses below the regression coefficients are z values.

### 4.3 Endogeneity test

The endogeneity treatment selects exchange rate *xr* and tariff *trf* as instrumental variables for the effect of export quality on innovation output to correct the endogeneity problem of the model. Exchange rates and tariffs affect export quality, but they do not directly affect firms’ innovation output, they can only further affect firms’ innovation output by affecting export trade. Model (1) shows the IV-2SLS regression results after including exchange rate and tariff as instrumental variables. The results show that after correcting the endogeneity problem of the model, the effect of export quality on firms’ innovation output is still significantly U-shaped. The results of the weak instrumental variable test show that the F-statistics are 144.337 and 68.505, respectively, and the Minimum eigenvalue statistic is 61.029, indicating that it is not a weak instrumental variable. The results of the over-identification test show that the Sargan and Basmann statistics are 0.0094 and 0.0091, respectively, and the corresponding p-values are 0.923 and 0.924, respectively, indicating that all instrumental variables are exogenously valid. Model (2) shows the IV-GMM regression results with exchange rate and tariff as instrumental variables. The results also remain robust and pass the weak instrumental variables test. The over-identification test shows a Hansen statistic of 0.013, corresponding to a p-value of 0.909, indicating that all instrumental variables are exogenously valid. Due to the missing values of innovation outputs for some of the samples, models (3) and (4) further use the Heckman two-stage method to correct for sample selection bias. The probability of firm innovation is first calculated and then this result is brought into the original regression equation. From model (3), the results of the LR test are significant at the 5% level, indicating the presence of sample selection bias, and the effect of export quality on innovation output remains a significant U-shaped relationship after the correction, and the significance is increased from 5% to 1%. The regression result of the model (4) shows that the IMR (lambda) is significant and the original regression model needs to be corrected and the result remains robust after correction. The specific regression results are shown in Table 5.

**Table 5:** Endogenous test.

	(1)	(2)	(3)	(4)
	IV-2SLS	IV-GMM	Heckman-MLE	Heckman-two step
***expqlt***	0.157** (0.072)	0.156** (0.063)	0.111** (0.047)	0.124** (0.055)
***expqlt*** ^***2***^	0.156** (0.069)	0.156** (0.067)	0.116*** (0.037)	0.125*** (0.043)
**lambda**			-0.694 (0.249)	-1.262** (0.638)
**Control Variables**	Yes	Yes	Yes	Yes
**Year FE**	Yes	Yes	Yes	Yes
**Industry FE**	Yes	Yes	Yes	Yes
**Province FE**	Yes	Yes	Yes	Yes
**Obs**	413	413	1811	1811
**LR test**			4.03**	
**R2**	0.400	0.400		
**Weak instrumental variables F**	144.337***	108.124***		
	68.505***	33.827***		
**Minimum eigenvalue statistic**	61.029	61.029		
**Overidentifying restriction**	0.0094	0.013		
	0.0091			

Note:*** p<0.01

** p<0.05

* p<0.1. All explanatory variables are ln*patents*. Values in parentheses below the regression coefficients are standard errors.

### 4.4 Heterogeneity analysis

#### 4.4.1 Property rights

The classification of company property rights can be categorized into two main groups: state-owned and non-state-owned. The sub-sample regression results presented in columns (1) to (3) demonstrate a statistically significant U-shaped association between export quality and the innovation output of non-state-owned firms. The inflection point of this relationship is observed at -0.2, with 183 observations falling on one side of the inflection point and 293 observations on the other side. There is no discernible impact observed in both the present and previous periods for state-owned enterprises.

One plausible explanation for these results is that state-owned enterprises (SOEs) are more susceptible to policy constraints and operate within non-market economic systems. Furthermore, due to the unique characteristics of their property rights, these entities often receive preferential treatment in terms of financial support, which can lead to reduced motivation for innovation. Additionally, they may exhibit a higher propensity for internal corruption and less efficient commercial operations. Consequently, the enhancement of export quality may not exert a substantial influence on the innovation of state-owned firms. This underscores the significance of improving the long-term and sustainable quality of export products, particularly for non-state-owned firms, including foreign-funded enterprises and private enterprises. The empirical findings suggest that while short-term quality enhancements may not positively impact firms’ innovation output in the current period, the long-term effects of improving export quality are more significant in terms of fostering innovation. Table 6 presents the results of a heterogeneity test conducted on property rights and industrial technology level, shedding light on the variations in the impact of export quality on innovation output across different types of enterprises and technological contexts.

**Table 6:** Heterogeneity test of property rights and industry technology level.

variable	(1)	(2)	(3)	(4)	(5)	(6)
	SOE	SOE	Non-SOE	High-tech	Low-and medium-tech	Low-and medium-tech
***expqlt***	-0.057 (-0.708)		0.088 (0.749)	0.083 (1.342)	-0.059 (-0.580)	
***expqlt*** ^***2***^	-0.565 (-0.122)		0.720** (2.218)	8.870** (2.150)	2.531 (0.446)	
***L*.*expqlt***		0.044 (0.921)				0.276 (1.335)
***L*.*expqlt*** ^***2***^		0.529 (1.329)				-0.471 (-0.085)
**Cons**	1.012*** (2.700)	0.185 (0.601)	1.716** (1.996)	3.021*** (8.505)	1.895*** (3.418)	4.650*** (5.157)
**Obs**	281	233	514	478	319	288
**Number of companies**	106	94	272	199	169	151
**Control variables**	Yes	Yes	Yes	Yes	Yes	Yes
**Year FE**	Yes	Yes	Yes	Yes	Yes	Yes
**Company FE**	Yes	Yes	Yes	Yes	Yes	Yes
**Industry FE**	Yes	Yes	Yes	Yes	Yes	Yes
**Province FE**	Yes	Yes	Yes	Yes	Yes	Yes
**Likelihood ratio test**	225.6	296.5	17.51	353.01	160.93	15.01

Note:*** p<0.01

** p<0.05

* p<0.1. Values in parentheses below the regression coefficients are z values.

#### 4.4.2 Industry technology level

The manufacturing industry can be categorized into two distinct technology levels: the high technology industry and the medium and low technology industry. The sub-sample regression results of model (4) indicate that in high-technology industries, the enhancement of export quality has a dual effect on the innovation output of manufacturing businesses. Initially, it hinders the innovation production, but afterwards, it stimulates it. The inflection point has been determined to be -0.4, with 209 observations preceding it and 269 observations following it. Models (5) and (6) demonstrate that there is no statistically significant impact of export quality on the innovation output of firms operating in medium- and low-technology industries.

The aforementioned findings suggest that there is a significant imperative to foster the enhancement of product quality within high-technology sectors. The lack of a statistically significant impact of export quality on the innovation output of medium and low-technology industries could potentially be attributed to the fact that these industries in China have achieved a high level of product quality across various dimensions as a result of the rapid development experienced during the past few decades of reform and opening up. Consequently, the influence of export quality on innovation may not have been observable within the timeframe of the sample period. Simultaneously, it is noteworthy that the gold content and incremental value derived through innovation within low-tech businesses are considerably lower in comparison to their high-tech counterparts. It is recommended that the emphasis on enhancing product quality be redirected towards high-tech industries, as this has a significant and favorable impact on the long-term innovation output of enterprises.

#### 4.4.3 Age of business

Firms can be classified into young and mature firms, which are determined by their duration of operation, the median age of the firms in the sample was used as the dividing criterion. The regression analysis of model (1) reveals a U-shaped association between export quality and the innovation output of new enterprises, with statistical significance at the 10% level. The regression findings presented in column (2) demonstrate a statistically significant U-shaped relationship between the lagged export quality of young enterprises and their innovation output. The regression coefficient is 0.997, which is deemed significant at the 1% level, the inflection point is located at -0.2, with 166 observations on the left side and 312 observations on the right side. The sub-sample regression results of model (3) and model (4) indicate that the coefficient of the squared term representing the impact of export quality (both in the current period and lagged one period) on mature businesses is positive, but it is not statistically significant.

It is evident that newly established enterprises, characterized by a relatively short period of establishment, should capitalize on the growth phase to enhance the quality of their exported products. Furthermore, these enterprises should demonstrate a long-term commitment to improving export quality and strategically leverage this period to enhance innovation output. Such endeavors are highly advantageous for the growth trajectory of young enterprises. However, in the case of well-established enterprises, the quality of exports does not have a substantial impact on the level of innovation output exhibited by these firms. One potential rationale for this outcome is that start-ups or nascent enterprises exhibit higher levels of innovation, greater motivation to innovate, heightened market survival pressures, and reduced reliance on prior exporting experiences compared to well-established, mature firms. Established firms with a longer history possess a comprehensive understanding of market dynamics, having acquired valuable insights into the intricacies of competition and the fluctuations of the external environment. Consequently, they have developed a repertoire of competitive advantages and possess the knowledge and strategies necessary to ensure organizational stability. Furthermore, the establishment of long-term business arrangements might result in a heightened state of route dependence. Additionally, an extended dependence on established export partnerships can impede the acquisition of diverse sources of innovation for well-established companies.

#### 4.4.4 Export destination

The sample is categorized into two groups: high-income countries and non-high-income countries. This categorization is determined by considering the proportion of OECD nations in the countries where exports are sent, with a threshold set at the median value. Based on the regression findings of the model (5), it is evident that the innovation output of firms engaged in exporting their products to high-income nations within the OECD exhibits an initial increase followed by a subsequent reduction in response to improvements in export quality. This pattern indicates the presence of an inverted U-shaped connection, specifically observed at a lag of three. The inflection point is determined to be at 0, with 92 observations on one side and 183 observations on the other side. The impact of export destinations on non-high-income countries is found to be statistically insignificant in both the current era and the lagged period.

One potential explanation for the observed regression outcome is that the high-income countries included in the study are predominantly developed nations. Moreover, a significant proportion of China’s general trade items shipped to these developed countries belong to industries characterized by low- and medium-technology sectors. These goods have a relatively low level of technical complexity. During the progressive enhancement of export quality among Chinese enterprises, when confronted with the initial sales and feedback from high-income developed countries, these enterprises undergo a rapid absorption, improvement, and learning process. Consequently, this leads to an early-stage growth in the performance of China’s enterprise innovation output. However, it should be noted that the potential for upgrading and improving the quality and technology of medium and low-tech industry products is limited. Once a certain level of quality is achieved, there will be a point at which further advancements become challenging. The rate of innovation output then diminishes when the export trade with industrialized nations reaches a state of equilibrium over a period of time. Conversely, China’s processing trade predominantly involves the exchange of medium and high-tech industry products with high-income developed nations. In the immediate timeframe, the implementation of production line equipment or technology may lead to an increase in innovative practices. However, over an extended period, it may be observed that processing trade does not yield a substantial positive effect on firm innovation. Hence, there exists a substantial inverted U-shaped correlation between the innovation output of Chinese businesses and the enhancement in the quality of exports to high-income nations. Table 7 displays the results of the heterogeneity test conducted on firm age and export destination.

**Table 7:** Heterogeneity test of firm age and export destination.

variable	(1)	(2)	(3)	(4)	(5)	(6)
	Young	Young	Mature	Mature	high-income	Non-high-income
***expqlt***	-0.011 (-0.217)		0.059 (0.926)			
***expqlt*** ^***2***^	5.533* (1.850)		5.961 (1.275)			
***L*.*expqlt***		0.054 (0.956)		0.042 (0.733)		
***L*.*expqlt*** ^***2***^		1.440*** (3.922)		0.673 (0.169)		
***L3*.*expqlt***					0.121 (1.643)	-0.052 (-0.772)
***L3*.*expqlt*** ^***2***^					-1.59*** (-3.103)	0.125 (0.241)
**Cons**	0.765** (2.394)	0.313 (0.893)	0.552 (0.942)	0.468 (0.891)	2.516*** (5.740)	3.247*** (7.163)
**Obs**	538	478	293	348	419	412
**Number of companies**	231	225	179	153	180	175
**Control variables**	Yes	Yes	Yes	Yes	Yes	Yes
**Year FE**	Yes	Yes	Yes	Yes	Yes	Yes
**Company FE**	Yes	Yes	Yes	Yes	Yes	Yes
**Industry FE**	Yes	Yes	Yes	Yes	Yes	Yes
**Province FE**	Yes	Yes	Yes	Yes	Yes	Yes
**Likelihood ratio test**	439.7	337.8	257.7	348.4	392.86	259.73

Note:*** p<0.01

** p<0.05

* p<0.1. Values in parentheses below the regression coefficients are z values.

#### 4.4.5 Region

The firm is categorized into two regions: the eastern region and the central and western regions. The analysis of model (1) and model (2) reveals a notable U-shaped association between the influence of export quality (current and lag period) and innovation output for enterprises operating in the eastern region. The regression analysis of model (3) indicates that there is no statistically significant relationship between export quality and firms’ innovation output for companies located in the central and western areas.

One potential explanation for this result could be that companies situated in the eastern region exhibit a greater capacity for learning, competition, and serve as notable representatives of China. Consequently, the innovative impact of export trade becomes effectively demonstrated, leading to an augmentation of firms’ innovative output through improvements in the quality of exported goods. Despite the fact that the effect of the central and western regions is not statistically significant, it is evident from the negative regression coefficient that businesses in these areas are still in the early stages of development. They have not yet acquired sufficient learning and absorption capabilities, resulting in comparatively weaker competitiveness when compared to enterprises in the eastern region. The challenge faced by participants in export trade lies in their inability to fully comprehend and assimilate the technological spillover generated during the trade process, thus hindering their capacity to significantly influence the innovative capabilities of firms and their innovation output.

#### 4.4.6 Trade mode

The mode of trade may be categorized into two main types: general trade and processing trade. However, due to the limited number of pure processing trade firms in the sample, the regressions are conducted by combining both pure processing trade enterprises and enterprises that have both modes of trade simultaneously. The regression results obtained from model (4) indicate that there is no statistically significant U-shaped relationship between export quality and innovation output for firms exclusively engaged in general trade. According to Model (5), there is a statistically significant positive relationship between export quality and innovation output at the 5% significance level. This implies that enterprises exclusively involved in general trade can enhance their innovation output by increasing the quality of their exports. Based on the regression outcomes of model (6) and model (7), it is evident that there exists a statistically significant U-shaped association between export quality and innovation output for firms engaged in processing trade. This relationship remains consistent across both the present period and the lagged period.

Enhancing the export quality of enterprises exclusively involved in general trade is expected to make a substantial beneficial impact on innovation output. However, the inclusion of enterprises engaged in processing trade instead results in a notable U-shaped association. The disparity in innovation output can be attributed to the presence of processing trade, which serves to inhibit enterprises’ ability to innovate. Enterprises that engage in both processing trade and general trade experience a notable decrease in their innovation output during the initial period. However, over time, it becomes evident that general trade serves as a means to harness the positive effects of innovation spillovers in export activities. Consequently, the adverse impact of processing trade on enterprise innovation output is temporary. International trade in goods and services conducted sustainably can generate positive social, economic, and environmental benefits. The results of the heterogeneity test for region and trading mode may be observed in Table 8.

**Table 8:** Heterogeneity test of region and trade mode.

variable	(1)	(2)	(3)	(4)	(5)	(6)	(7)
	Eastern	Eastern	Midwestern	General trade	General trade	Including processing trade	Including processing trade
***expqlt***	-0.122 (-0.896)					0.018 (0.296)	
***expqlt*** ^***2***^	6.670** (2.083)					1.150*** (2.740)	
***L*.*expqlt***		0.047 (0.911)	-0.298 (-1.174)	0.086* (1.821)	0.099** (2.444)		-0.0009 (-0.160)
***L*.*expqlt*** ^***2***^		0.105*** (2.685)	-0.144 (-0.828)	-2.071 (-0.601)			1.132*** (2.687)
**Cons**	2.291*** (6.098)	0.505 (0.581)	3.176** (2.536)	0.569 (1.590)	0.566 (1.582)	0.917*** (2.694)	0.921*** (2.675)
**Obs**	631	560	183	443	399	389	378
**Number of companies**	284	269	84	242	257	235	229
**Control variables**	Yes	Yes	Yes	Yes	Yes	Yes	Yes
**Year FE**	Yes	Yes	Yes	Yes	Yes	Yes	Yes
**Company FE**	Yes	Yes	Yes	Yes	Yes	Yes	Yes
**Industry FE**	Yes	Yes	Yes	Yes	Yes	Yes	Yes
**Province FE**	Yes	Yes	Yes	Yes	Yes	Yes	Yes
**Likelihood ratio test**	611.62	56.40	38.23	306.9	306.7	311.7	275.7

Note:*** p<0.01

** p<0.05

* p<0.1. Values in parentheses below the regression coefficients are z values.

## 5. Conclusion

This study does an empirical analysis to examine the influence of export quality on the level of innovation production across manufacturing enterprises in China. The aforementioned findings can be summarized as follows: Initially, it is observed that the impact of export quality on the innovation output of Chinese manufacturing businesses exhibits a noteworthy U-shaped relationship throughout the complete sample. The association between enhanced export quality and the number of patent applications for inventions and utility models exhibits a U-shaped pattern, but the influence on design patents is shown to be statistically insignificant. There is a notable U-shaped correlation between export quality and both domestic and international patent applications, with the impact on domestic patent applications being more immediate. Furthermore, upon doing heterogeneity analysis, it has been determined that the quality of China’s manufacturing exports has a notable U-shaped impact on the innovation output of non-state-owned firms, while its effect on state-owned enterprises is not significant, a notable U-shaped correlation in the high-technology sector, but no statistically significant impact is observed in the medium- and low-technology sectors. The innovation output of new firms has a lagged and statistically significant U-shaped effect, while the influence on mature enterprises is found to be not significant. There exists a lagged inverted U-shaped association between the innovation production of firms and the export destination country’s income level, specifically in the case of high-income countries. However, no substantial impact on the innovation output is observed when the destination country is a non-high-income country. The innovation output for firms in the eastern region exhibits a notable U-shaped impact, whereas the impact on the central and western regions is not statistically significant. The analysis also reveals a notable U-shaped correlation between firms involved in processing commerce and their innovation production. Additionally, a favorable impact is observed exclusively for enterprises engaged in general trade on their innovation output.

The results of this empirical study suggest that there is a need to improve the quality of their export products in a long-term and sustained manner for Chinese manufacturing firms, especially non-state-owned firms, young firms, high-tech firms, and firms in the eastern region. The state should formulate a series of quality standards for the dynamic development of different industries and use dynamic quality standards to require enterprises to continuously improve the quality of their products, as well as to actively protect the long-term and continuous innovation needs of these enterprises.

This paper conducts an empirical study, building upon the research findings of Lin and Lu [29], Chen and Reyes [9], and Larsen [33], among others. The study unveils diverse research conclusions, revealing a U-shaped relationship between product quality and the innovation output of enterprises. Furthermore, it offers valuable insights into the applicability of its findings. Owing to data constraints, Future research could extend the sample duration, delve into the mechanisms through which export quality impacts innovation, or consider adjustments at the sample level, such as provincial-level, product-level, or industry-level product quality.
